# The relationship between behavioral language laterality, face laterality and language performance in left-handers

**DOI:** 10.1371/journal.pone.0208696

**Published:** 2018-12-21

**Authors:** Lise Van der Haegen, Marc Brysbaert

**Affiliations:** Department of Experimental Psychology, Ghent University, Ghent, Belgium; Washington University in Saint Louis School of Medicine, UNITED STATES

## Abstract

Left-handers provide unique information about the relationship between cognitive functions because of their larger variability in hemispheric dominance. This study presents the laterality distribution of, correlations between and test-retest reliability of behavioral lateralized language tasks (speech production, reading and speech perception), face recognition tasks, handedness measures and language performance tests based on data from 98 left-handers. The results show that a behavioral test battery leads to percentages of (a)typical dominance that are similar to those found in neuropsychological studies even though the incidence of clear atypical lateralization (about 20%) may be overestimated at the group level. Significant correlations were found between the language tasks for both reaction time and accuracy lateralization indices. The degree of language laterality could however not be linked to face laterality, handedness or language performance. Finally, individuals were classified less consistently than expected as being typical, bilateral or atypical across all tasks. This may be due to the often good (speech production and perception tasks) but sometimes weak (reading and face tasks) test-retest reliabilities. The lack of highly reliable and valid test protocols for functions unrelated to speech remains one of the largest impediments for individual analysis and cross-task investigations in laterality research.

## Introduction

Speech production is one of the most clearly lateralized functions in the human brain. Control is dominated by the left hemisphere (LH) in 88% to 96% of right-handers [1–3]. Cases of atypical speech lateralization in the right hemisphere (RH) or bilateral patterns can be found in large-scale neuroimaging studies with functional transcranial Doppler Sonography or functional Magnetic Resonance Imaging (fMRI), but they require a serious investment before a group of 20 participants is obtained. Clear RH dominance is mainly found in left-handers with estimates ranging from 6.5% to 27% compared to 0 to 4% in right-handers [1–4]. Particularly in healthy student populations the prevalence of RH speech dominance seems to be limited to some 10% of the left-handers [2–4].

Because of their larger variability, left-handers should be the first choice of study in experiments that aim to study correlations of functions. Surprisingly, that is not the case. Most studies are either limited to right-handers [5] or include a small number of left-handers (because the prevalence of left-handedness is limited to some 10–14% of the population). A small number of left-handers is a problem because the majority of left-handers is still LH dominant (see also[6, 7]) so that the number of participants with non-LH dominance in an unselected sample can be expected to be very small (some 20% of 10% or 2 out of 100 participants). Indeed, [8] showed that left-handedness by itself does not predict a reversed ear asymmetry in the dichotic listening task; such reversal was only observed when the lefthanders were limited to a group with established atypical speech dominance. The typical right ear advantage (REA) pointing at LH speech perception dominance was observed both in left- and right-handers, but an atypical left ear advantage (LEA) was only present in a subgroup of left-handers previously identified as being RH speech dominant.

Language processing and specifically speech laterality based on fMRI measurements is an interesting starting point to classify subjects in (a)typical categories for brain organization because of its strong lateralization and known incidence of RH dominance by large scale neuroimaging studies [9]. It is however also important to know how other functions are lateralized in left-handers to investigate cognitive relationships in healthy individuals. Hemispheric specialization characterizes many functions such as emotional processing, spatial attention, memory etc. [10]. The present article takes a first step by relating indices of face and language processing, together with different measures of handedness, to the laterality of language (speech production, reading, and speech perception).

The present study contributes to the existing literature in five ways. First, we provide the distribution of lateralization indices for the three main functions of language in the same sample of participants. Previous (neuroimaging) studies combined maximally two language-related tasks in right-or left-handers (e.g.[4, 11]). They concluded that language subprocesses can occasionally lateralize to different hemispheres or, more likely, to a different degree. In particular, evidence for unilateral control was stronger for the inferior frontal gyrus involved in speech production than for the ventral occipito-temporal region involved in reading. The current study additionally looked at the correlations with speech perception.

Second, all current tasks are behavioral computer tasks. Lateralized behavioral tasks have extensively been used in the past [12–15]. For vision, the visual half field (VHF) task is used most often. In this task better performance to stimuli presented in the left visual half field (LVF) indicates RH functional dominance and better performance to stimuli in the right visual half field (RVF) indicates LH dominance, because the optic fibers partially cross on their way from the eyes to the brain. We used methodologically optimized versions of the VHF task following guidelines from [16–17]. A new lateralized paradigm was developed to test reading lateralization (see below). For auditory modality, dichotic listening is the preferred technique. We used the standardized version of the Bergen group, which has been used in many research projects (e.g.[18]).

Behavioral tasks are interesting, because they require fewer resources than neuroimaging studies. They are particularly interesting for the initial screening of participants with potential atypical brain dominance [4]. Computer tasks are less time-consuming, expensive and restrictive for participant inclusion than neuroimaging techniques (e.g. they do not suffer from the problems of irremovable ferromagnetic material in the participants, noisy environment, and claustrophobia often encountered in fMRI research). We therefore wanted to explore whether a combination of behavioral tasks could provide us with consistent individual lateralization patterns that can have predictive value for the relationship between lateralized neural functions.

Behavioral tasks are unlikely to be specific enough for high-stake assessment (e.g., to determine speech lateralization in patients who have to undergo brain surgery), but they can serve as a quick first screening. In particular, we want to know whether VHF tasks can give a quick first indication of the lateralization of a wide variety of functions related to language, memory, emotional processing, face processing and so on. For this we need validated tasks, preferentially versions that do not take too much time. [19] gives an example of a smartphone app based on dichotic listening, which can be used for screening ear preferences in large groups of the population.

The third contribution of this study is that we added a face VHF task to the language tasks. This allowed us to further examine the many-to-many view on brain organization proposed by [20]. According to this view, face processing elicits a LVF/RH advantage in people who have learned to read because face processing and reading compete for neural resources in the ventral visual cortex (see for example [13] for first behavioral evidence in right- and left-handers). As a result, learning to read (which critically depends on the language dominant hemisphere) pushes face processing to the less taxed homolog brain region in the non-dominant hemisphere. Given the available preliminary evidence for this view we aimed to validate the contralateral dominance of reading and face processing in our large sample of left-handers containing a wide variety of lateralization patterns.

The fourth contribution of the present study is that we related laterality information to language performance, as measured with tests of naming speed and vocabulary size. Mixed results have been reported in the literature. [21] argued that the issue is not yet resolved because researchers rarely measure overlapping functions. However, even in studies that carefully matched lateralization function and performance test, inconsistent results have been reported. [14] for example used word and face VHF tasks and reported an inverted U-shaped curve, with optimal performance in the case of a bilateral VHF advantage and worse performance with more extreme LVF and RVF advantages. In contrast, [22] used data from the Bergen dichotic listening database and reported a U-shaped curve, with better performance for clear right or left ear advantages than for cases with no clear asymmetries. [23] also found a U-shaped curve when relating speech production fMRI LIs to a dozen of verbal and spatial performance tests. This study included a high number of left-handers from the BIL&GIN dataset [24], so that more variability was present in the participants. An aphasia patient study by [25] confirmed the finding of better language performance with more extreme lateralization indices in the case of rapidly progressing brain lesions. Their patients were all right-handed and showed LH lateralization in a verb generation task. Optimized lateralization thus seems to differ across language processes according to these studies. In the current study we further investigated the relationship between language laterality and performance by comparing LIs from behavioral tasks in a large left-handed sample.

Finally, the interpretation of correlations between laterality indices critically depends on the reliabilities of the indices. Two indices will not correlate if they are not reliable. Therefore, absence of correlation cannot be interpreted as evidence for independence of function unless the indices are shown to be reliable. The best way to assess the reliability of an index is to test it twice. For a well-designed behavioral lateralization task performance should vary more between participants than within participants tested twice. Lateralization indices in the sample should in addition have a broad distribution range to be able to represent individual variation [26]. We therefore invited 50 of our 100 left-handed participants for a second session one week after the first to redo the language and face test battery, so that we could evaluate test-retest reliability.

To meet the above aims, the following tasks were included. A first set consisted of two lateralized language tasks validated by [4] as a measure of speech production laterality. In these VHF tasks, participants are asked to name words (task 1) or pictures (task 2). Different words/pictures are presented in LVF/RVF and participants have to name the word/picture indicated by a centrally presented arrow as fast and accurately as possible. The LVF/RVF advantages correlated up to *r* = .76 with fMRI LIs in a word generation task (i.e. mentally think of as many words as possible starting with a target letter) in the study of [4]. Everyone who named RVF stimuli at least 25 ms faster than LVF stimuli was confirmed to be LH speech dominant in fMRI and all but one participant with an LVF advantage of at least 60 ms turned out to be atypically RH lateralized.

A lateralized lexical decision task based on [27] was developed as a behavioral predictor for reading lateralization (task 3). In that study, the authors showed that participants with typical language dominance named words faster when they fixated the first letter of a word than when they were forced to look at the last letter. The optimum for RH dominants lied more towards the word end. Words fixated at the first letter are transmitted almost entirely to the LH whereas words fixated at the last letter initially go to the RH because of the partial crossing of optic fibers. This pattern is part of the optimal viewing position effect (OVP; [28]) and indicates that there is no bilateral representation of the fovea but that letters in central vision are contralaterally sent to the brain instead [27]. An advantage of the OVP technique is that the words can be presented in central vision, rather than in parafoveal vision, where visual acuity is lower (and words are more difficult to identify). In the current lexical decision task, we presented six-letter words and pseudowords that could be fixated either at the first of last letter because naming latency differences between laterality groups were largest for this word length in [27].

The fourth task was the dichotic listening paradigm developed at Bergen University (e.g.[18]). We only used the non-forced condition of the paradigm, in which participants are asked to report which of two binaurally presented sounds they heard best or first. More reports from the left ear point at a RH dominance for auditory speech perception, whereas more reports from the right ear point at an LH dominance [29]. We chose to double the number of trials in the standard Bergen version from 36 to 72 and to skip the forced conditions in which participants have to report the stimuli from one ear and ignore the other side, because the forced conditions serve as an attention control for which we did not expect any differences between LH and RH dominant participants (confirmed in unpublished data from [8] who reported clear left/right ear advantages in RH/LH speech dominants respectively in the unforced condition and overall left/right ear advantages in the left/right forced conditions).

In addition to the language tasks, we also presented a face VHF task to the participants. It was based on [13, 20, 30], in which it was argued that word and face processing compete with each other because of overlapping brain tissue. As a result, [30] predicted stronger RH laterality of face perception when the fusiform gyrus in the LH is increasingly used for reading. In the paradigm of [13], a target face is presented centrally, followed by a face in the LVF or RVF. Participants are asked to indicate whether the two faces are the same or not by pressing buttons. We slightly adjusted the paradigm by using another face database [31], aligning fixation and stimulus presentation durations with the other VHF tasks we ran, and adding a face to the non-target side in order to avoid attentional biases. In [13] the VHF face task resulted in a group-level LVF advantage in terms of accuracy and reaction times in right-handers (N = 24) and also shorter reaction times for LVF stimuli in left-handers (N = 24). The same sample showed RVF/LH advantages in a word version of the paradigm. In our study, we wanted to explore how the distribution of VHF asymmetries looked like for a large group of left-handers containing individuals with atypical language (in particular reading) lateralization.

Handedness was measured in three ways. [32] found reduced LH story listening lateralization in participants with left-handers among their first-degree relatives and/or weak manual preferences as measured with the Edinburgh Handedness Inventory [33]. So, we included both the Edinburgh Handedness Inventory and a questionnaire about left-handedness among relatives in our test battery. In addition, we added a finger tapping task in which the participants were asked to press a button as many times as possible with their left or right indices during ten trials of ten seconds each (as in [34] cited in [24]).

The final part of our test battery consisted of three short language proficiency tests. Reading speed for words and pseudowords were measured with the Dutch één-minuut-test (EMT; one-minute-test; [35]) and the Klepel [36] in which participants named as many words or pseudowords as possible within one or two minutes respectively. These tests were included as brief indicators of reading ability because [37] showed that scores on a word identification test similar to the one-minute-test predicted more efficient eye movement behavior (e.g. reduced fixation durations) in sentence reading. [38] also showed that these tests made the biggest difference between control and dyslexic readers. We added the Lextale vocabulary test to the two naming speed tests [39]. In this test, participants are given a list of 40 words and 20 nonwords and they have to indicate which words they know (the nonwords are used to correct for guessing). We used Lextale, because [40] related higher scores on this questionnaire to asymmetries in a VHF lexical decision task in bilinguals. The current study did not take into account bilingualism even though most Flemish students can be considered as late bilinguals as they are taught French from fifth grade in primary school and English from second grade in high-school. The Lextale mainly served as a standard vocabulary test that can be related to differences in language proficiency.

## Method

### Participants

One hundred participants studying at Ghent University or a higher education school in Ghent were recruited via a website from the Faculty of Psychology and Educational Sciences where students can sign up for experiments in exchange for course credits (1^st^ bachelor students Psychology or Physical Education and Movement Sciences; N = 68) or money (N = 32). Mean age was 19.63 years (Range 17–34, SD 3.12). Sixty-nine participants were female. We did not balance the number of male and female participants because this study focused on the relationship between behavioral laterality tasks. Post-hoc t-tests between males and females for the reaction time and error rate lateralization indices based on the VHF and DL results reported below did not reveal any significant sex differences (*p*s > .24), except for the error lateralization indices in the face task [t(93) = 2.31, *p* < .02] with males showing more extreme RH dominance than females. The language performance neither showed significant differences between males and females (*p*s > .18).

Eligibility criteria to participate were: Being left-handed (i.e. report to at least write with their left hand, no other handedness tests were run on beforehand to ensure a wide variability even if that meant that a few participants turned out to be ambidexter in the handedness measurements below), having Dutch as mother tongue, having normal or corrected-to-normal vision, no prior participation in laterality studies and not having any hearing or reading disorders. We only tested students who reported to be left-handed in order to increase variability in the laterality tasks. Fifty students were retested with the same language and face laterality tasks one week after their first participation in order to obtain reliability measurements for all tasks. They were told that the experiment consisted of two sessions, but only heard at the beginning of the second session that they would be tested with the same tasks twice in order to minimize familiarity effects. Each session took 1.5 to 2 hours. All tasks were run in the same order for all participants and sessions in order to exclude order effects between the (a)typical/bilateral groups. Participants could take a break whenever they wanted for as long as they wanted (most breaks lasted a few minutes). One participant was excluded from all analyses because he reported having dyslexia at the end of the second session; another one was excluded because she had insufficient knowledge of Dutch to complete the language tasks. This brings the total number of participants tested to 98. The study was approved by the Research Council Flanders and the ethical committee of the Faculty of Psychology and Educational Sciences (Ghent University). All research was conducted according to the principles expressed in the Declaration of Helsinki. Participants signed a written informed consent prior to the start of the experiments.

### Handedness measurements

Students were allowed to participate if they reported to always write with their left hand. Three other handedness measurements were included. First, the twelve items of the widely used Edinburgh Handedness Inventory [33] could result in a score between -100 (complete left-handedness) to +100 (complete right-handedness) by applying the formula

((L−R)/(L+R))*100 (1)

with L/R being the number of left/right preferences and items with a strong preference receiving doubled weight. The Edinburgh Inventory was extended with the [41] questionnaire so that eyedness, earedness and footedness preferences were also measured with four items each. Second, familial sinistrality was questioned because having first-degree left-handed relatives has been shown to influence functional language asymmetry for story listening [32]. The number of left-handed parents, siblings or children was recalculated as a percentage for the current analyses. Finally, lateralized manual performance was tested in a finger tapping task (cfr. [23]), in which participants were asked to press a button on a Cedrus RB-730 response box as many times as possible during 10 seconds. They did this five times with the index finger of each hand, taking breaks in between as long as they wanted, starting with their dominant left hand, alternating between hands after each block and holding their hands in a similar position (i.e. with their wrist on the table). A lateralization index was calculated with formula (1), L/R being the total number of finger taps made with the left/right hand respectively.

### Language laterality tasks

#### Speech production: Picture and word visual half field (VHF) task

Stimuli. The picture and word VHF tasks were adopted from [17] and were the same as the Dutch versions used in [4]. In brief, the picture task consisted of five symmetrical line drawings (a boat, a book, a house, a lamp and a star) that could be presented in the LVF and RVF. The word task contained 96 Dutch three-letter words and 96 Dutch four-letter words. They were combined in target-filler pairs with targets and fillers having the same length, being both a noun or adjective, not starting with the same letter and being pairwise matched on summated type bigram frequency, log frequency per million and number of neighbors in the CELEX database (*p*s > .40; [42]). All stimuli can be found in the supplementary materials of [4].

Design. All targets could be presented in the LVF or RVF, always accompanied by another stimulus in the opposite visual field. This bilateral presentation avoided laterality effects due to attentional biases [16]. Each of the five line drawings in the picture VHF task was presented together with one of the other four drawings either in the LVF or the RVF. All possible pairs were repeated four times resulting in a total of four experimental blocks of 40 trials separated by a break. A practice phase of eight trials made the participant familiar with the task. The target words in the word VHF task were shown once in the LVF and once in RVF, always combined with the same filler item. There were three blocks of 64 trials, again separated by breaks and preceded by a practice phase of 16 trials. Both tasks additionally contained 10% randomly presented trials with a centrally displayed digit between 1 and 9, presented for only 80 ms and followed by a 80 ms mask as a motivation to keep fixating the screen center. Participants named 99,3% of the digits correctly in the picture VHF task (only 14 subjects made errors with a maximum of 3/16) indicating that participants did fixate the screen center when asked. The percentage of correctly reported numbers in the word VHF task was not registered due to a programming error, but there were no signs during testing that they performed less than in the picture task. [4] in addition reported that fixation control with or without an eye-tracking device only minimally influences the results of these two tasks.

Procedure. Each trial started with a central fixation cross for 500 ms. Stimuli pairs were then bilaterally presented for 200 ms with a central arrow pointing in the direction of the target to be named as fast and accurately as possible. A 200 ms mask consisting of random lines (picture VHF task) or #### (word VHF task, Courier New font size 15) prevented participants from fixating the stimulus in case they made an eye movement towards the target. As in [4], participants were seated at a distance of approximately 60 cm from the screen. All stimuli were presented at least 1.6 degrees of visual angle away from the screen center meaning that they were located in parafoveal vision, even though foveal vision is also found to be split (e.g. [27]). After the mask, the fixation cross was shown until the participant made a naming response. The experimenter finally coded the response as being correct, incorrect or a voice key failure.

#### Reading: Lateralized optimal viewing position (OVP) task

Stimuli. The OVP task contained 150 words and 150 pseudowords selected from the Dutch Lexicon Project [43]. They were all six letters long because this word length showed the clearest laterality effect in [27]. All words were nouns consisting of one or two syllables (Mean: 1.86) with a mean raw Celex frequency of 975 (Range: 5–8793, SD: 1622), mean sum of non-positional trigram frequencies of 25 717 (Range: 2129–122 464, SD: 20 592) and mean Coltheart N (i.e. number of words differing one letter) of 3.26 (Range: 0–16, SD: 3.46). Half of them were fixated at the first letter and half at the sixth letter. These two lists were matched on the abovementioned variables (*p*s > .76). Pseudowords were pairwise matched with the words on trigram frequency and Coltheart N (*p*s > .43), and across both fixation position lists (*p*s > .57).

Design. Words and pseudowords were only presented once, either in the LVF or RVF when being fixated at the last or first letter respectively. Each cell of the within-subjects Word type (word vs. pseudoword) x VHF (LVF vs. RVF) design contained 75 trials that were randomly presented and divided across six blocks. Four words and four pseudowords that were not part of the experimental list served as practice trials. Digits between 1 and 9 were again included as fixation control in 10% of the trials and had to be judged as being odd or even by pressing buttons (Mean accuracy 95.0%, SD: 5.47).

Procedure. A trial looked as follows: (1) two vertically aligned lines were presented at the screen center for 400 ms and the participant was asked to fixate between them from the moment they appeared; (2) A stimulus was presented for 150 ms between the fixation lines; (3) Participants bimanually pressed two inner/outer buttons of a Cedrus RB-730 device with their index/middle fingers to indicate whether the target was a word/pseudoword respectively while the fixation lines remained on the screen; (4) An intertrial interval of 1500 ms. The screen stood at a reading distance of approximately 60 cm. Stimuli were in Courier New font size 15, such that each letter subtended 0.3° and the outmost letter was 1.65° away from the central fixation lines.

#### Speech perception: Dichotic listening (DL) task

Stimuli. The DL task was similar to the standard paradigm developed at the University of Bergen, Norway (e.g. [18]). The six stop consonants /b/, /d/, /g/, /p/, /t/ and /k/were combined with the vowel /a/ based on recordings by a native Dutch speaker (as in [8]). Thirty consonant-vowel syllable pairs were formed by a simultaneous auditory presentation of two different stimuli, one to each ear. Six homonym pairs were added as a control that the participants could discriminate the stimuli (and they could; Mean correct homonym identification: 88.6%; SD: 10.37).

Design & procedure. The current task only included the non-forced condition of the standardized paradigm in which the participant was asked to say out loud the syllable (s)he heard best or first. They were informed that they might hear two different sounds through the headphone but were only allowed to give one response. All participants were tested with the same computer with the speakers at the same loudness level. Participants were asked to give a sign if the stimuli were too loud or too silent but nobody did. All possible consonant-vowel syllables were visually shown beforehand. The number of trials was doubled to 72 in this study with a break halfway the experiment. Consonant-vowel syllables lasted around 350 ms. The intertrial interval was 4000 ms.

#### Face laterality task

Stimuli. Forty colored photos of faces in front view were adopted from [31]. They all had a neutral expression. Hair, glasses etc. were removed. Half of them were female. Masks were created by scrambling the photos with a Fourier phase randomization procedure to preserve global low-level characteristics of the stimuli (for more details see [31]). There was a grey background behind the faces; the masks were of the same size without any background. The screen background was white throughout the experiment.

Design. Participants were asked to judge whether a probe face was the same as the face thereafter presented in the direction of a central arrow. There were two within-subject factors: VHF (LVF vs. RVF) and Same/Different (Same: same probe and target vs. Different1: same probe and filler vs. Different2: different probe, target and filler). There were 80 Same-trials and 80 Different-trials (i.e. 40 Different1 and 40 Different2 trials to avoid responses being based on identification of the filler). All faces could serve as a probe, a target or a filler. They were paired with a different face across conditions. The targets appeared once in the LVF and once in the RVF. The mask was always the same scrambled version of the preceding face. The participant could take three breaks in-between. Twenty trials preceded the experiment as a practice phase. Digits from 1 to 9 were again briefly presented in 10% of the trials as fixation control (Mean accuracy: 94.0%, SD: 7.64).

Procedure. Each trial started with a centrally presented fixation cross for 500 ms. The probe image was shown for 1500 ms, followed by a 700 ms blank screen. A second 500 ms fixation cross introduced the target-filler pair that was only presented for 200 ms, immediately followed by the 200 ms masks. The inner boundaries of the photos were 1° away from the screen center where an arrow pointed in the direction of the target during the face presentations. A central fixation cross replaced the arrow during the mask and remained on the screen until the participant pressed two inner/outer buttons on a Cedrus RB-730 device to indicate that the target was the same as/different from the probe respectively. The intertrial interval was 500 ms.

### Language performance tasks

Three short tests were administered to obtain a score for language-related abilities. The een-minuut-test (EMT; i.e. one-minute-test; [35]) measures word reading speed by letting participants read out loud as many words as possible in one minute. The list contains 116 words in increasing difficulty. A score is calculated as the number of correctly read words minus the number of errors made without the participant correcting him/herself. In the current study, the score was based on words read in 45 instead of 60 seconds, because 12 participants needed less than a minute to complete the list (that is often used in dyslexia research e.g. [38]). The mean score was 81 (SD: 12). A second test was called *De Klepel* [36] and is very similar to the one-minute-test, except that pseudowords instead of words must be read out loud. Participants get two minutes in the standard test, but here we chose a time limit of one minute to avoid ceiling effects. Mean score on this test was 62 (SD: 11). Third, the Lextale questionnaire was included as a vocabulary test [39]. Participants had to mark 60 items as being an existing Dutch word or not without time pressure. Scores are calculated as

numberof((wordscorrect/40*100)+(nonwordscorrect/20*100))/2 (2)

Participants scored on average 90 on the Lextale (SD: 6).

## Results

The raw data of the VHF tasks and dichotic listening were first cleaned before we ran the main analyses. This involved the exclusion of the following trials for reaction time (RT) analyses: errors (Picture VHF: 2.5%, Word VHF: 19.4%, OVP: 9.9%, Face VHF: 24.0%), voice key failures (i.e. no trigger, triggered by environmental noise, participant saying uh, coughing etc.; Picture VHF: 14.6%, Word VHF: 3.5%), RTs less than 200 ms or greater than 1500 ms (Picture VHF: 0.5%, Word VHF: 0.0%, OVP: 0.3%, Face VHF: 10.7%) and for the remaining trials latencies above/below 2.5 SDs from the participant's mean RT (Picture VHF: 1.9%, Word VHF: 8.5%, OVP: 2.0%, Face VHF: 0.6%). Only words and no pseudoword conditions were taken into account in the OVP task RT and accuracy analyses. In the face VHF task, only Same responses and no Different conditions were included. A few data files were lost due to technical problems (e.g. computer failure, a badly timed fire drill etc.; Picture VHF: N = 1, Word VHF: N = 1, OVP: N = 2, DL: N = 1, Face VHF: N = 1, One-minute-test: N = 4, Klepel: N = 1). Finally, seven data points were excluded upon visual inspection of the trade-off between individual mean RT and accuracy (see S1 Table; Picture VHF: N = 1, OVP: N = 3, Face VHF: N = 3). Data points were only removed for the outlier task. Other data from these participants were kept, except for the same task in the second session. One more data point in the Word VHF task was removed because the participant only named 3% trials correctly in the LVF. Table 1 shows the mean (and SD) RTs and accuracy scores for the LVF and RVF in the first session of each task together with the effect sizes of the VHF difference (based on the means, SDs and correlation of both VHFs; [44]) because they may be of interest for future meta-analyses. Analyses reported below were based on LIs calculated with formula (1) to enhance the comparability across tasks (with for DL right ear reports–left ear reports instead of LVF-RVF for the VHF tasks with reaction times so that a higher score always represents a higher LH advantage), unless otherwise mentioned. All individual raw and LI data can be found in the S1 Table.

**Table 1:** Mean reaction times, accuracy scores (in % errors) and their standard deviations in the left and right visual field of the visual half field tasks. The mean (and standard deviation) lateralization index is calculated based on formula 1 (see main text). Cohen’s d is provided as effect size measure of the visual half field differences and is based on the means, standard deviation and correlation of both visual half fields. For the dichotic listening task, the means and standard deviations of left and right ear matches are reported instead of reaction times.

**Task**	**Reaction times**			
	**Mean RT (SD) in LVF**	**Mean RT (SD) in RVF**	**Mean LI (SD)**	**Cohen's d**
Picture VHF	732 (97)	711 (103)	1.54 (3.29)	0.47
Word VHF	490 (124)	452 (113)	3.75 (6.38)	0.57
OVP	548 (107)	519 (101)	2.74 (3.05)	0.81
DL	22 (6.57)	26 (7.18)	3.53 (12.80)	0.31
Face VHF	498 (146)	550 (155)	-5.27 (8.74)	-0.53
**Task**	**Accuracy scores**			
	**Mean error (SD) in LVF**	**Mean error (SD) in RVF**	**Mean LI (SD)**	**Cohen's d**
Picture VHF	3.45 (4.90)	2.18 (3.00)	25.22 (56.71)	0.34
Word VHF	30.18 (19.79)	10.59 (9.97)	49.77 (33.71)	1.12
OVP	8.81 (5.40)	6.14 (3.74)	14.68 (37.49)	0.51
DL	NA	NA	NA	NA
Face VHF	17.58 (14.40)	27.16 (15.50)	-22.39 (37.97)	-0.58

SD = standard deviation; LVF/RVF = left/right visual field respectively; VHF = visual half field; LI = lateralization index; OVP = Optimal Viewing Position; DL = Dichotic Listening.

### Prevalence of (a)typical lateralization per task

**Fig 1:**
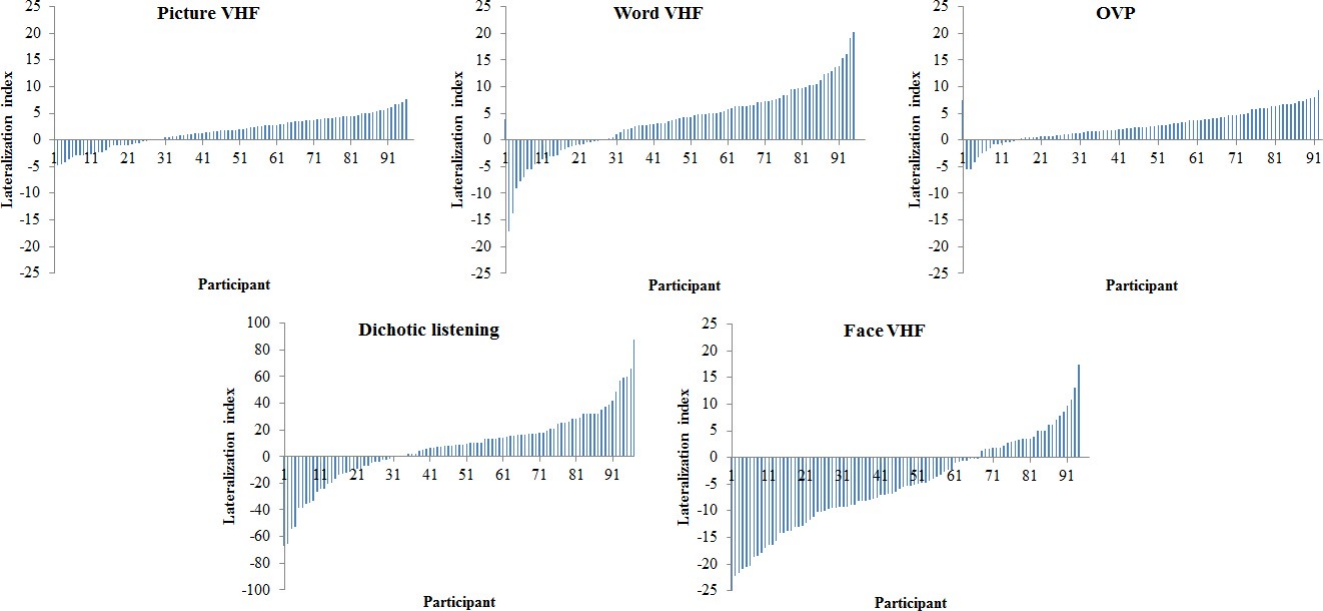
Reaction time lateralization indices for the laterality tasks. Reaction time lateralization index (LI) distributions for the picture visual half field (VHF), word VHF, Optimal Viewing Position (OVP), dichotic listening and face VHF tasks. LIs are sorted from most negative to most positive scores per task. Note that only the dichotic listening chart has a deviating scale on the y-axis to improve visibility because LIs were based on left/right ear matches and not reaction times.

Fig 1 shows the reaction time LI distributions of the participants for the picture VHF, word VHF, OVP, DL and face VHF tasks.

**Fig 2:**
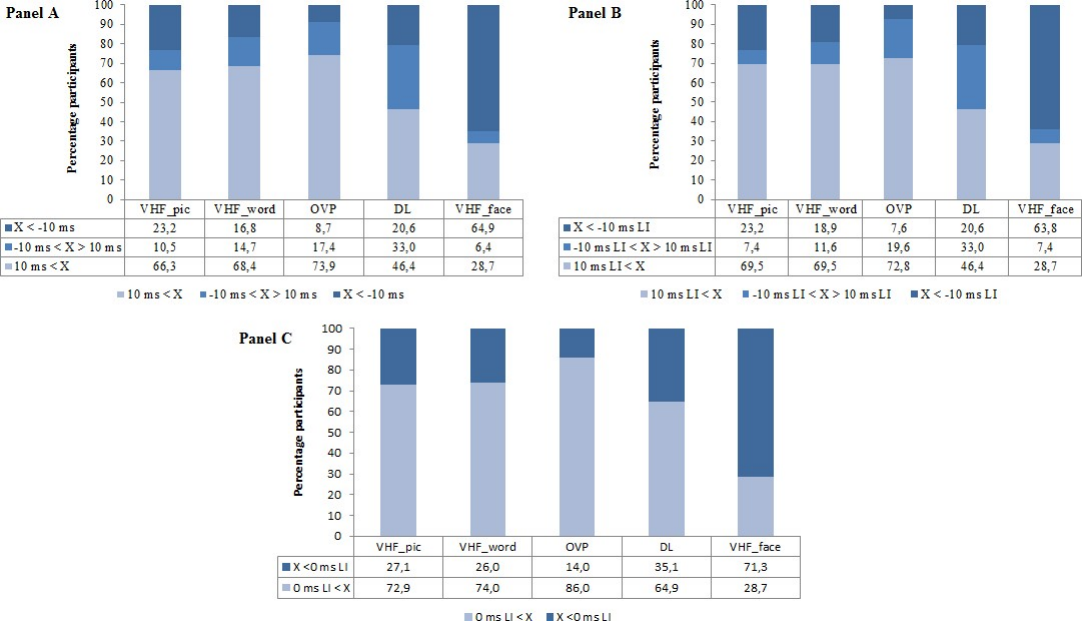
Percentage participants categorized as showing typical, bilateral or atypical lateralization based on their reaction times. Reaction time distributions expressed as percentages and divided in a typical, bilateral and atypical category for the picture visual half field (VHF), word VHF, Optimal Viewing Position (OVP), Dichotic Listening (DL) and face VHF tasks. Panel A classifies the participants based on raw differences between the left and right visual field or ear. The data in Panels B and C are based on lateralization indices (LIs) that represent a mean reaction time difference of 10 ms (same values as in Panel A for the DL task) and 0 ms respectively.

Fig 2 shows the same information when the laterality indices are categorized as typical, bilateral, and atypical. Panel A is based on individual raw differences of mean RT LVF–mean RT RVF. For the DL task, which does not require the participant to respond as fast as possible, the LI based on accuracy scores was taken because DL results are usually reported in this form in the literature. Negative differences below -10 ms were considered as an indication of RH dominance, differences above 10 ms as an indication of LH dominance and values in between as an indication for an unclear difference or bilateral lateralization pattern. The percentages below the graph confirm the expected LH dominance for language and RH dominance for face processing in the majority of participants, ranging from 65 (Face) to 74% (OVP). Only the DL task classified slightly less than half of the participants (46%) as clearly LH dominant with this LI = 10 criterion. Clear atypical dominance is present in 9% (OVP) to 29% (face VHF). The prevalence of non-clear typical dominance (i.e. unclear patterns and clear RH dominance together) is around 30% for most tasks, except for the DL task that elicited more bilateral patterns (in 33%) than the other tasks. Note however that the testing procedure and scoring of DL deviates from the other tasks which may lead to a different distribution across laterality types. We chose to report the raw data in this way, because it is the most commonly used method and thus enhances comparison with previous studies. DL LI scores are often cut off at a value of 0 instead of 10 which in this sample results in 65% participants with a typical right ear/LH advantage.

Panel B shows the distribution based on LI scores for each task. We further chose to calculate an LI limit for the three categories based on the current dataset, i.e. LI scores that represent a mean RT difference of 10 ms between the VHFs in our sample (absolute LI boundary value for Picture VHF: 0.71, Word VHF: 1.13, OVP: 0.97, Face VHF: 1.03). A 10 ms/items boundary stays an arbitrary choice, but recalculating the LIs in this way optimizes the comparability across tasks within this study. Moreover, an LI of 1 equaled a 14 ms (Picture VHF), 9 ms (Word VHF), 11 ms (OVP) and 10 ms (Face VHF) RT VHF difference indicating that an overall 10 ms boundary is a reasonable choice for this dataset. This is confirmed by the fact that the percentages in each laterality category barely change from Panel A to B. A difference of 10 reported consonant-vowel syllables from the left and right ear in the DL equaled an absolute LI boundary value of 21.1. This again clearly deviates from the other values because the maximum difference is 60 items compared to 1300 ms in the other tasks and seems not appropriate given that this results in only 23% participants with a clear LH dominance. This is unrealistic given that we know that speech perception lateralizes to the LH in the majority of people. It is however impossible to determine an LI limit that is comparable to the LI boundaries in the RT experiments. Panel B therefore kept the original 10 LI boundary for DL. In addition, we show the prevalence of (a)typical lateralization per task based on a LI cut-off score of 0 in Panel C. We believe leaving out bilateral patterns is the only way to compare laterality tasks using a different method given the difficulty of determining universal LI limits. A classification based on a LI 0 ms/0 ear reports difference mainly increased the number of typical LH dominants in the DL and OVP tasks. This brings the overall image of laterality categories to a comparable level across all included tasks, i.e. typical patterns ranging from 65% (DL) to 86% (OVP) and atypical patterns ranging from 14% (OVP) to 35% (DL). The OVP lexical decision task elicited the highest percentage of typical LH lateralization but bear in mind that no less than 20% participants fell in the bilateral category in Panel B. Finally, if we count how many participants belong to the same laterality category for all task LIs based on a 0 LI cut-off score 26% was typically lateralized overall and none was atypical for all tasks. These percentages increase when only taking the language tasks into account without the face VHF (Typically LH dominant: 39%, atypically RH dominant: 5%). These percentages are still far below what can be expected based on previous studies and the percentages per task in Fig 2, especially in the typical group.

### Relationship of degree of lateralization between behavioral laterality tasks

Exploring Pearson correlations between the LIs of the laterality tasks, handedness measurements and language performance tests can shed further light on how these variables are linked in left-handers on a continuous scale. These results tell more about the influence of degree of lateralization than the categorical approach reported in 3.1. In this section, we will present correlations that test the relationship between the abovementioned variables based on RT LIs and accuracy LIs together with correlations between RT and accuracy for each task where applicable, and scatter plots to contrast two tasks against each other.

#### Reaction time, accuracy and reaction time-accuracy correlations

Table 2 displays the Pearson correlations between all variables tested, with the LIs based on RT for the VHF laterality tasks. First, the language tasks correlate significantly and positively with each other as expected even though the correlations are rather low (between *r* = .26, *p* < .05 for VHF picture-DL and *r* = .38, *p* < .001 between VHF word-OVP), except for the VHF picture with OVP task (*r* = .17, *p* = .12). The VHF face task does not correlate significantly with any other variable (*p*s > .09). With respect to handedness, no correlation was found between the overall Edinburgh Inventory score and any of the lateralized tasks (*p*s > .30), nor with the language performance one-minute, Klepel or Lextale tests (*p*s > .11) and not even with finger tapping or familial sinistrality (*p*s > .21). The total handedness score only correlated positively with the separately questioned lateral preferences for hand, ear, eye and foot use (*r*s between .37 and .94, *p*s < .001). Note however that this sample only contained left-handers to increase variability in laterality so the handedness scores represent weak to strong preferences without covering both negative left-handed and positive right-handed scores. The one-minute and Klepel reading tests strongly correlated (*r* = .69, *p* < .001), but otherwise the performance tests did not show any relationship with other variables except for a few rather weak correlations (EMT-VHF picture: *r* = .25, *p* = .02; Klepel-OVP: *r* = .22, *p* = .04; Lextale-Hand: *r* = .21, *p* = .04; Lextale-Eye: *r* = .33, *p* < .01). A polynomial curve such as a U-shaped relationship in which bilateral VHF or ear differences go together with a lower performance would however not become visible in the Pearson correlations. We therefore provide the scatter plots contrasting the laterality and language performance tests in S1 Fig. As can be seen, there is no polynomial relationship either despite quite some variability in the EMT and Klepel scores (but a limited range in the Lextale vocabulary test).

**Table 2:** Pearson correlations between all variables tested, with the lateralization indices based on *reaction times* for the visual half field tasks and left/right ear matches for the dichotic listening task.

	Vhf Picture	Vhf Word	OVP	DL	Vhf Face	EHI	Hand	Ear	Eye	Foot	Finger-tapping	Familial sinistrality	EMT	Klepel	Lextale
**Vhf Picture**	1														
**Vhf Word**	,342**	1													
**OVP**	,165	,382**	1												
**DL**	,262*	,294**	,356**	1											
**Vhf Face**	,121	,120	-,093	-,094	1										
**EHI**	-,008	,064	,109	,051	-,082	1									
**Hand**	-,018	,100	,119	,066	-,134	,943**	1								
**Ear**	,078	,091	,218*	,011	-,033	,370**	,245*	1							
**Eye**	,015	,143	,203	,218*	-,172	,459**	,450**	,256*	1						
**Foot**	,002	,117	,143	,128	-,179	,783**	,779**	,367**	,447**	1					
**Finger-tapping**	-,133	-,135	,166	,051	-,119	,128	,199	-,056	,087	,085	1				
**Familial sinistrality**	,073	-,005	-,001	-,012	,173	-,009	-,005	,043	-,002	-,090	,005	1			
**EMT**	,252*	,017	,085	,067	-,004	-,106	-,087	-,078	,046	-,050	-,043	,012	1		
**Klepel**	,197	,011	,219*	,057	-,003	-,023	-,023	-,024	,057	-,106	-,049	,060	,689**	1	
**Lextale**	,067	,020	,031	,135	-,144	,164	,211*	,020	,327**	,119	-,031	,022	,105	,173	1

VHF = visual half field: DL = dichotic listening; OVP = Optimal Viewing Position; EHI = Edinburgh Handedness Inventory; EMT = One-minute-test.

** denote significant correlations at the 0.01 level (2-tailed)

* denote significant correlations at the 0.05 level (2-tailed).

Table 3 contains the correlations based on accuracy in the VHF laterality tasks. The included DL LIs are the same as those in Table 2. Only two positive significant accuracy correlations were observed: Between DL and VHF word (*r* = .31, *p* < .01) and between DL and OVP (*r* = .26, *p* < .05; all other *p*s > .08). Four weakly significant, positive correlations were found between the questionnaire data of lateral preferences and laterality tasks (Ear-VHF picture: *r* = .21, *p* = .05; Ear-VHF word: *r* = .26, *p* = .01; Eye-VHF word: *r* = .24, *p* = .02; DL-Eye: *r* = .22, *p* = .03) and three stronger correlations with the VHF face accuracies (Total EHI: *r* = -.29, *p* < .01; Hand: *r* = -.32, *p* < .01; Foot: *r* = -.31, *p* < .01).

**Table 3:** Pearson correlations between all variables tested, with the lateralization indices based on *error rates* for the visual half field tasks and again left/right ear matches for the dichotic listening task.

	Vhf Picture	Vhf Word	OVP	DL	Vhf Face	EHI	Hand	Ear	Eye	Foot	Finger-tapping	Familial sinistrality	EMT	Klepel	Lextale
**Vhf Picture**	1														
**Vhf Word**	,093	1													
**OVP**	,056	,185	1												
**DL**	-,051	,314**	,260*	1											
**Vhf Face**	-,087	,070	-,090	-,146	1										
**EHI**	,110	,189	,088	,051	-,291**	1									
**Hand**	,030	,169	,108	,066	-,318**	,943**	1								
**Ear**	,205*	,261*	,138	,011	-,015	,370**	,245*	1							
**Eye**	,002	,239*	,110	,218*	-,169	,459**	,450**	,256*	1						
**Foot**	,189	,115	,184	,128	-,307**	,783**	,779**	,367**	,447**	1					
**Finger-tapping**	-,033	-,035	,083	,051	,068	,128	,199	-,056	,087	,085	1				
**Familial sinistrality**	-,090	,024	-,124	-,012	,017	-,009	-,005	,043	-,002	-,090	,005	1			
**EMT**	,071	-,035	,005	,067	,047	-,106	-,087	-,078	,046	-,050	-,043	,012	1		
**Klepel**	-,005	,169	,048	,057	,005	-,023	-,023	-,024	,057	-,106	-,049	,060	,689**	1	
**Lextale**	,050	,078	,085	,135	-,152	,164	,211*	,020	,327**	,119	-,031	,022	,105	,173	1

VHF = visual half field: DL = dichotic listening; OVP = Optimal Viewing Position; EHI = Edinburgh Handedness Inventory; EMT = One-minute-test.

** denote significant correlations at the 0.01 level (2-tailed)

* denote significant correlations at the 0.05 level (2-tailed).

Third, Table 4 presents the correlations between RTs and accuracy for the laterality VHF tasks (not for DL as there is no time pressure for participants to respond) to investigate the speed-accuracy trade-off in each task and evaluate whether both latency and accuracy data could serve as an LI. The RT and accuracy LIs did significantly correlate in all four tasks despite big differences in accuracy rates (e.g. on average only 2.5% errors made in the VHF picture task compared to 24.0% errors in the VHF face task, see section 3; *r*s between .32 and .59, *p*s < .01).

**Table 4:** Pearson correlations between reaction times and error rates for the four visual half field tasks. The speed-accuracy trade-off correlations per task are highlighted in bold.

	Vhf Picture Error	Vhf Word Error	OVP Error	Vhf Face Error
**Vhf Picture RT**	,324^**^	,227*	,126	-,221*
**Vhf Word RT**	,023	,585^**^	,297^**^	,017
**OVP RT**	-,040	,349^**^	,315^**^	-,128
**Vhf Face RT**	,135	,187	-,052	,369^**^

RT = reaction time; VHF = visual half field; OVP = Optimal Viewing Position.

** denote significant correlations at the 0.01 level (2-tailed)

* denote significant correlations at the 0.05 level (2-tailed).

#### Scatter plots contrasting two laterality tasks

**Fig 3:**
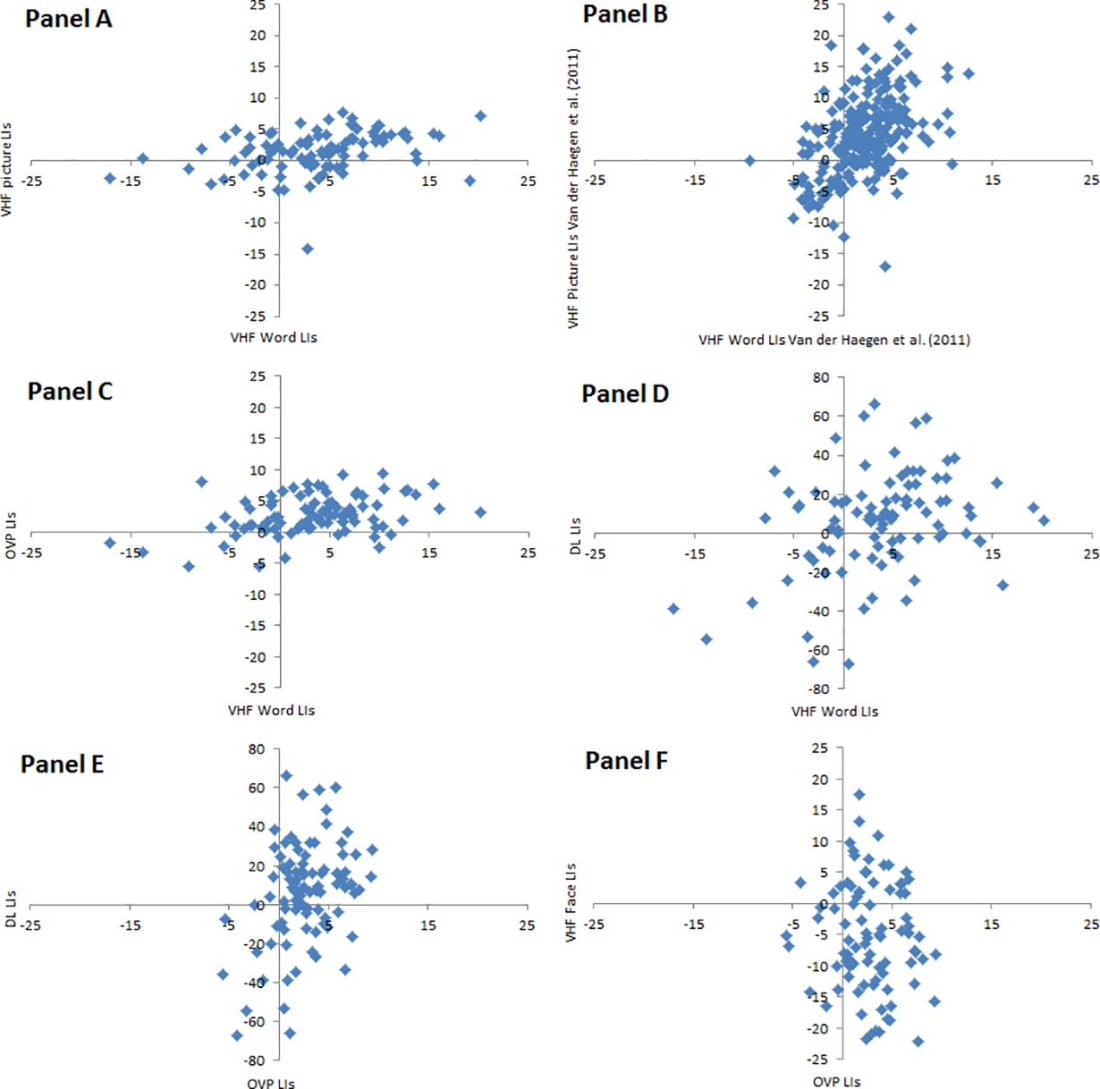
Scatter plots contrasting two tasks. Data points represent individual lateralization indices (LIs) based on reaction times. VHF = Visual Half Field; OVP = Optimal Viewing Position; DL = Dichotic Listening. Note that only the dichotic listening chart has a deviating scale on the y-axis to improve visibility because LIs were based on left/right ear matches and not reaction times.

Fig 3 contains scatter plots showing the most interesting LI contrasts between two tasks based on RTs for the VHF tasks. Panel A contrasts the two speech production VHF tasks. Most LIs fall in the upper right quarter of the scatter plot, i.e. the quarter with participants that have a positive LI for both the word and picture task as expected based on the literature and the significant correlation reported in 3.2.1 (*r* = .34, *p* < .01). We plot data from the same tasks tested in 250 left-handers in [4] in Panel B for a comparison with the current study. For this study, the VHF picture and word LIs recalculated with formula (1) correlated with a value of *r* = .46, *p* < .001. 77% participants fell in the LH/RH category for both tasks when taking an LI cutoff of 0 compared to 63% in the current study. The LIs correlated most highly with LIs measured in an fMRI word generation task when only taking into account participants that showed clear (i.e. with a 10 ms VHF cut-off) and consistent VHF asymmetries (i.e. *r* = .76 and .74, *p*s < .001 for the picture and word task respectively). Indeed, 85% fell in the same laterality category when taking LI limits based on an average 10 ms VHF difference in [4]. A similar criterion in the current study also increases the number of consistent participants to 75%. The remaining panels of Fig 3 will mainly be evaluated based on an LI cutoff score of 0 because of the abovementioned complexity to calculate LI limits in the DL task.

Panels C and D contrast the VHF word task against OVP and DL respectively. We only include the VHF word and not picture results because both tasks measure the same underlying function (i.e. speech production), correlate (see 3.2.1 and previous paragraph) and the word task is slightly more stable than the picture task (see 3.3). A cutoff value 0 classified 74% in the LH or RH laterality category for both the word and OVP task with as expected the majority of participants in the upper right quarter of Fig 3C. Leaving out participants with a bilateral VHF difference based on a 10 ms LI again helped to increase the incidence of consistent participants to 85%. Panel D (VHF word vs. DL) only placed 66% of the participants in the upper right (consistent LH dominance) or lower left (consistent RH dominance) panel. Combining OVP and DL 0 LI cutoff classifications resulted in 71% consistent participants, mainly driven by the many LH dominant patterns in the OVP task. Finally, typical or atypical lateralization for reading in the OVP and face processing in the VHF face task was found in 63% of the participants. This contrast shows the least consistent patterns, presumably again because of the large majority of participants showing an RVF advantage in the OVP task in addition to a low reliability of the currently used face task (see 3.3). Using a stricter 10 ms LI cutoff did not improve the consistency this time as expected because only 7% VHF face participants had an unclear bilateral VHF difference as reported in 3.1.

### Test-retest reliability of laterality tasks

A laterality score from one test can maximally correlate with a laterality score from another test to the degree that it correlates with itself. Tables 5 (RTs) and 6 (accuracy) provide the correlations between the first and second session for each laterality task and the finger tapping task (only included in Table 6), tested in about half of the participants. The RT LIs of all dominant hemisphere tasks correlated more across the two sessions than the correlations between tasks described in 3.2.1, ranging from *r* = .49, *p* < .01 (OVP task) to *r* = .83, *p* < .001 (VHF word). In contrast, the laterality indices from the VHF face task did not correlate across the two sessions (*r* = .22, *p* = .12), pointing at a weak reliability of the task in its current form.

**Table 5:** Pearson correlations between the lateralization indices based on *reaction times* for session 1 (variables in rows) and session 2 (variables in columns, indicated by task name 2). The test-retest correlations per task are highlighted in bold.

	VHF Picture 2	VHF Word 2	OVP 2	DL 2	VHF Face 2
**VHF Picture**	,769^**^	,358*	,399^**^	,146	-,050
**VHF Word**	,464^**^	,829^**^	,437^**^	,173	-,116
**OVP**	,317*	,259	,489^**^	,199	-,209
**DL**	,338*	,425^**^	,312*	,671^**^	-,040
**VHF Face**	,165	,274	-,082	-,330*	**,223**

**VHF** = Visual half field; OVP = Optimal Viewing Position; DL = Dichotic Listening (using indices based on left/right ear matches).

** denote significant correlations at the 0.01 level (2-tailed)

* denote significant correlations at the 0.05 level (2-tailed).

**Table 6:** Pearson correlations between the lateralization indices based on *error rates* for session 1 (variables in rows) and session 2 (variables in columns, indicated by task name 2). The test-retest correlations per task are highlighted in bold. Note that correlations with fingertapping lateralization indices are only included in this table, not in Table 5.

	VHF Picture 2	VHF Word 2	OVP 2	DL 2	VHF Face 2	Finger-tapping 2
**VHF Picture**	**,065**	-,037	,032	-,181	-,144	,212
**VHF Word**	,319*	,505^**^	,271	,159	-,329*	-,157
**OVP**	,198	-,121	,384^**^	,265	-,080	,089
**DL**	,076	,333*	,119	,671^**^	-,149	,094
**VHF Face**	-,006	,108	-,062	-,113	,347*	-,077
**Finger-tapping**	,168	-,029	,256	,218	,008	,876^**^

**VHF** = Visual half field; OVP = Optimal Viewing Position; DL = Dichotic Listening (using indices based on left/right ear matches).

** denote significant correlations at the 0.01 level (2-tailed)

* denote significant correlations at the 0.05 level (2-tailed).

The accuracy reliability correlations in Table 6 were somewhat lower but still highly significant for the VHF word, OVP, DL and finger tapping task (Range: *r* = .38, *p* < .01 for OVP to *r* = .88, *p* < .001 for finger tapping that was not included in the previous paragraph). The test-retest correlation for accuracy in the VHF picture task was not significant (*r* = .07, *p* = .67) but note that only 2.5% errors were made on average. The accuracy LIs of the VHF face task did correlate in contrast to the latency LIs, though still rather weakly (*r* = .35, *p* = .02), presumably because of the high error rates (on average 24.0%) that can be informative to which side face processing lateralizes.

As in 3.2.2 we will also express the stability of the tasks in terms of percentage participants that belong to the same laterality category based on 10 ms LI without bilateral patterns for all tasks except DL and a 0 cutoff LI value for all tasks including DL. The percentages for the 10 ms LIs were 85 (VHF picture), 90 (VHF word), 91 (OVP) and 68 (VHF face). In other words, even though the correlated LIs were significant but moderate to high, categorizing participants as being clearly LH or RH dominant was rather stable across two sessions except for the VHF face task (a 10% error LI limit for accuracy in the face task resulted in 79% stable classifications). The percentages based on a 0 cutoff LI score were 83 (VHF picture), 83 (VHF word), 82 (OVP), 78 (DL) and 67 (VHF face; 69% for accuracy).

## Discussion

The current study provides for the first time an overview of the distribution of lateralization indices for language processes (VHF word and picture task for speech production, lateralized lexical decision task for reading and dichotic listening for auditory speech perception) and face processing in a large sample of left-handers. The test battery only included behavioral computer tasks that are easy to run and half of the subjects performed the tasks twice to assess the test-retest reliability. Language proficiency and handedness measurements were included as well.

Left-handers were chosen because of their larger variability. This makes them a more interesting group to study atypical dominance and correlations between functions. Indeed, if none of the participants have atypical dominance (which may very well be the case for a group of righthanders tested), a correlation between two laterality indices says nothing about the co-lateralization of functions (as they are all lateralized to the same hemisphere), but only informs us about the correlation of the lateralization strengths of these (see also [45]).

Dividing participants in a typically and atypically lateralized group based on positive and negative LIs resulted in about 70% typical dominance and 30% atypical dominance in the VHF and DL tasks. These percentages agree reasonably well with the often cited incidence of about 27% atypical speech dominance in left-handers found by [1] who also used a cut-off value of 0 for their word generation task in functional Transcranial Doppler Sonography. fMRI large-scale studies introduced a third category with participants who show an unclear dominance pattern [2–4]. They only report 6.5 to 10% clear speech RH dominance in left-handers. The current tasks however resulted in about 20% clear atypical lateralization when leaving out bilateral patterns based on a 10 ms LI difference (except for the 8% in the OVP lexical decision task). This leads to two interesting points of discussion. First, behavioral VHF tasks seem to overestimate the prevalence of clear atypical dominance at the group level compared to fMRI studies. The method chosen seems to mainly affect the prevalence of atypical patterns. Second, the importance of choosing an appropriate way to define categories based on LIs should not be underestimated (cfr. the discussion of LIs that should be independent of arbitrarily chosen thresholds in fMRI laterality studies; [46]) even if that means that the same LI calculation method cannot be used for all tasks in the same test battery. The dichotic listening task in particular seemed to evoke more bilateral patterns when using a 10 LI boundary. This does not mean VHF tasks are better predictors of neural language lateralization because the DL task has been convincingly validated to reflect speech perception lateralization (e.g. [47]) when using a 0 cut-off value. It stresses that behavioral tasks using a different method should be treated in a different way when creating laterality categories. Asking participants to report which sound they heard best does not involve an accuracy-speed trade-off and LI boundaries are restricted by the number of items instead of milliseconds. The lower percentage of typical DL dominance may also be related to the fact that the task assesses phonological processing in speech perception (which may be more bilaterally distributed) rather than lexical and semantic processing (which may be more lateralized, as argued by [48]).

Our data further showed that LIs based on reaction times and accuracies do not lead to major differences. The picture of prevalence of (a)typical lateralization remained the same. The relationships between functions also showed the same global pattern for reaction time and accuracy LIs: language laterality tasks correlated with each other, but not with the face task (meaning that in contrast to [13] no evidence could be found for the many-to-many view on brain organization which may be due to the low reliability of the current VHF face task, see below). There were also significant correlations between reaction time and accuracy LIs within the tasks that measured both variables (i.e. VHF word, picture, face and OVP). Of course, reporting all results should be encouraged in studies but the large differences in task difficulty with performances at ceiling level in the VHF picture task and only two significant correlations between the language tasks based on accuracy LIs seem to suggest that using latency LIs for further analyses optimize the comparability across VHF studies. RT-based indices are best when accuracy is high, whereas accuracy-based measures are optimal when accuracy is low.

We also notice that in our study language lateralization did not correlate with naming speed (words in the one-minute-test, pseudowords in Klepel) or vocabulary knowledge (Lextale). This may partly be due to the low reliability of the laterality indices (the language tests are known to have reliabilities over .8), although it may be interesting to keep in mind that previous studies described in the Introduction also found limited or mixed relationships between degree of lateralization and performance (e.g. [14, 22, 23]). This is an issue that is likely to require a study with high power or a meta-analysis to be settled. It would also be interesting to see whether age or educational level influences the correlation between language lateralization and language performance. We could not test this in the current study because these two factors were too homogeneous in our sample and almost all participants scored very high on the speeded naming and vocabulary tests. More variability due to age or educational level could reveal a closer relationship with language lateralization. The finding that the handedness measures (Edinburgh Inventory combined with the Porac and Coren questionnaire, familial sinistrality and finger tapping) did not correlate with language laterality is interesting as well, although it should be kept in mind that all our participants were left-handed. This limits the range of the handedness variable. Still, it is interesting to know that for lefthanders there is little covariation between the degree of laterality and language performance.

Despite the abovementioned expected laterality patterns at the group level, the results also made clear that a behavioral laterality test battery does not yield consistent patterns across tasks at the individual level. If all five tasks were considered with a 0 LI cut-off score, only 27% of the participants would be typical dominant overall. One can debate over the exact prevalence, but it is known that the majority of people is typically lateralized. In other words, even methodologically well-controlled behavioral tasks cannot replace the more accurate laterality estimates measured in neuroimaging yet. This is particularly true for the indices close to 0. In [4] we showed that RT-based VHF differences become more useful for interpretation when they are larger, because the vast majority of participants with an RVF/LVF advantage of over 25/60 ms on the VHF naming tasks were LH/RH dominant for speech in the scanner respectively. As a result, behavioral test batteries do not seem to be useful (yet) in clinical settings. Even for a quick pre-surgery screening testing multiple cognitive functions the individual patterns are too inconclusive at this moment. In the future, patients with brain damage could reveal unique information about the relationship between functions when tested with optimized test batteries, because this population also shows a wide variety in laterality indices. Similar to [25] who reported better language performance with more extreme lateralization, we could for example speculate that performances are better if a clear separation of language and face lateralization is preserved because that is the standard organization in healthy subjects.

The low individual stability may in part be explained by the significant but sometimes rather low test-retest reliability of the tasks. The VHF picture (r = .77) and word task (r = .83) are close to the clinical standard of excellent reliability of .8, and the DL task had a good reliability above .6. The reliabilities of the new paradigms we tried out show that they are open to improvement with r = .49 for the OVP task and even non-significant reliabilities for the VHF face task (r = .22 for the RT-based laterality index, r = .35 for accuracy; see for example [49–50] for other lateralized face paradigms). In particular, finding reliable lateralized non-speech tasks turns out to be a challenge.

In order to optimize paradigms we think it is important researchers develop detailed protocols and share their findings, even when these are less neat and convincing than they had hoped. [51] advised to administer more trials in VHF tasks to obtain more reliable VHF asymmetries and thus more individual stability. They asked their participants to name five series of 100 four- and five-letter words. However, the time needed for such a test would cancel most of the benefits behavioral tasks have over neuroimaging studies (even in terms of money if one must pay for the tester and the testing room). We could not foresee this, but the lack of reliability turned out to be the main limitation of the current study. If reliability had been better, we could have concluded more about the relationship between functions tested by computer tasks. We advise future studies to first optimize the individual tasks in terms of test-retest reliability before implementing them in a test battery. Our experiences with the face recognition task further point to the importance of following existing procedures (protocols) strictly. Every change introduced may have (usually negative) consequences on the reliability and should be tested in advance.

Linking low individual consistency to the test-retest reliability raises a final interesting point of discussion. [52] recently argued that cognitive tasks robust at the group level may differ from tasks useful to reveal individual differences. Experimental psychologists try to find stable, reproducible effects at the group level with a minimum of variance between participants (therefore often excluding left-handers, see above), whereas psychologists interested in individual differences require tasks with a maximum of systematic variance between individuals. These may be different tasks, with test-retest reliability a particularly important aspect for research into interindividual differences. Stable individual differences are also important in studies looking for the degree of correlation that can be expected between tasks. If individuals cannot be distinguished on one dimension measured by task A, there cannot be a correlation with another dimension measured by task B ([53], cited in [52]).

Alternatively, it could be that behavioral laterality tasks show low intercorrelations because large networks in the brain contribute to task performance (measured with speed or accuracy). This may be too blunt an instrument to measure the laterality of functions that are limited to a very small brain region. Laterality of faces, for example, is limited to a tiny region in the fusiform area [49, 54]. It may be that this area contributes only a small part of the VHF differences observed in a behavioral laterality task. For such functions, neuroscientific techniques with a higher resolution are a better measure.

Should we conclude that behavioral tasks have to be banned from laterality research? Not yet. Behavioral laterality tasks based on speech production can serve as a valid, quick screening method for (a)typical speech dominance [4, 49] and are stable enough to reveal overall laterality patterns when the tasks are methodologically optimized. There may be other tasks of similar use, which we have not found yet. For such tasks it is not so much important that they show a big difference between left and right hemisphere processing (e.g., a big VHF advantage), but that they are related in a reliable and valid way to individual differences in brain laterality. It will be interesting to see whether such behavioral tasks can be found. Inspiration from neuroscientific findings is likely to be important in this search.

## Supporting information

S1 Table The supporting information file VanderhaegenBrysbaert_supplementarymaterials_individualdata.xlsx contains a table with raw individual data of all tasks mentioned in the main text. I.e.: Participant numbers corresponding to the raw data files on OpenScienceFramework DOI10.17605/OSF.IO/Y28HQ; sex; age; mean reaction times and error rates from the left and right visual field in the picture, word, face visual half field task and optimal viewing position task along with their corresponding lateralization indices; number of left and right ear reports in the dichotic listening and the corresponding lateralization indices; global Edinburgh Handedness Inventory scores and hand, ear, eye, foot scores; percentages familial sinistrality; total number of finger taps with the left and right hand and the corresponding lateralization indices; One-minute-test, Klepel and Lextale scores.
VHF = visual half field; RT = reaction time; LVF = left visual field; RVF = right visual field; LI = lateralization index; OVP = optimal viewing position; DL = dichotic listening; EHI = Edinburgh Handedness Inventory.
(XLSX)

S1 Fig Scatter plots contrasting the laterality indices (i.e. from the word, picture and face visual half field task, optimal viewing position task and dichotic listening) and language performance scores (i.e. from the One-minute test, Klepel and Lextale). VHF = visual half field; LI = lateralization index; OVP = optimal viewing position; DL = dichotic listening; EMT = One-minute-test.
(TIF)

## References

[1] Knecht S, Drager B, Deppe M, Bobe L, Lohmann H, Floel A, et al Handedness and hemispheric language dominance in healthy humans. Brain. 2000;123:2512–8. 11099452 10.1093/brain/123.12.2512

[2] Mazoyer B, Zago L, Jobard G, Crivello F, Joliot M, Perchey G, et al Gaussian Mixture Modeling of Hemispheric Lateralization for Language in a Large Sample of Healthy Individuals Balanced for Handedness. Plos One. 2014;9(6). 10.1371/journal.pone.0101165 PMC4076312 24977417

[3] Pujol J, Deus J, Losilla JM, Capdevila A. Cerebral lateralization of language in normal left-handed people studied by functional MRI. Neurology. 1999;52(5):1038–43. 10102425 10.1212/wnl.52.5.1038

[4] Van der Haegen L, Cai Q, Seurinck R, Brysbaert M. Further fMRI validation of the visual half field technique as an indicator of language laterality: A large-group analysis. Neuropsychologia. 2011;49(10):2879–88. 10.1016/j.neuropsychologia.2011.06.014 21708178

[5] Willems RM, Van der Haegen L, Fisher SE, Francks C. On the other hand: including left-handers in cognitive neuroscience and neurogenetics. Nature Reviews Neuroscience. 2014;15(3):193–201. 10.1038/nrn3679 24518415

[6] Allendorfer JB, Hernando KA, Hossain S, Nenert R, Holland SK, Szaflarski JP. Arcuate fasciculus asymmetry has a hand in language function but not handedness. Hum Brain Mapp. 2016. 10.1002/hbm.23241 PMC4988400 27144738

[7] Ocklenburg S, Beste C, Arning L, Peterburs J, Güntürkün O. The ontogenesis of language lateralization and its relation to handedness. Neurosci Biobehav Rev. 2014;43:191–8. 10.1016/j.neubiorev.2014.04.008 24769292

[8] Van der Haegen L, Westerhausen R, Hugdahl K, Brysbaert M. Speech dominance is a better predictor of functional brain asymmetry than handedness: A combined fMRI word generation and behavioral dichotic listening study. Neuropsychologia. 2013;51(1):91–7. 10.1016/j.neuropsychologia.2012.11.002 23149380

[9] Cai Q, Van der Haegen L. What can atypical language hemispheric specialization tell us about cognitive functions? Neuroscience Bulletin. 2015;31(2):220–6. 10.1007/s12264-014-1505-5 25822216 PMC5563700

[10] Ocklenburg S, Hirnstein M, Beste C, Gunturkun O. Lateralization and cognitive systems. Front Psychol. 2014;5:1143 10.3389/fpsyg.2014.01143 25339936 PMC4189433

[11] Van der Haegen L, Cai Q, Brysbaert M. Colateralization of Broca's area and the visual word form area in left-handers: fMRI evidence. Brain Lang. 2012;122(3):171–8. 10.1016/j.bandl.2011.11.004 22196742

[12] Bless JJ, Westerhausen R, von Koss Torkildsen J, Gudmundsen M, Kompus K, Hugdahl K. Laterality across languages: Results from a global dichotic listening study using a smartphone application. Laterality. 2015;20(4):434–52. 10.1080/1357650X.2014.997245 25588000 PMC4425226

[13] Dundas EM, Plaut DC, Behrmann M. Variable left-hemisphere language and orthographic lateralization reduces right-hemisphere face lateralization. J Cogn Neurosci. 2015;27(5):913–25. 10.1162/jocn_a_00757 25390197

[14] Hirnstein M, Leask S, Rose J, Hausmann M. Disentangling the relationship between hemispheric asymmetry and cognitive performance. Brain Cogn. 2010;73(2):119–27. 10.1016/j.bandc.2010.04.002 20472334

[15] Klichowski M, Kroliczak G. Numbers and functional lateralization: A visual half-field and dichotic listening study in proficient bilinguals. Neuropsychologia. 2017;100:93–109. 10.1016/j.neuropsychologia.2017.04.019 28414092

[16] Bourne VJ. The divided visual field paradigm: Methodological considerations. Laterality. 2006;11(4):373–93. 16754238 10.1080/13576500600633982

[17] Hunter ZR, Brysbaert M. Visual half-field experiments are a good measure of cerebral language dominance if used properly: Evidence from fMR1. Neuropsychologia. 2008;46(1):316–25. 10.1016/j.neuropsychologia.2007.07.007 17716695

[18] Hugdahl K. Dichotic listening in the study of auditory laterality In: Hugdahl K., Davidson R.J., editors. The asymmetrical brain. Cambridge, MA: MIT Press; 2003.

[19] Bless JJ, Westerhausen R, Arciuli J, Kompus K, Gudmundsen M, Hugdahl K. "Right on all Occasions?"—On the Feasibility of Laterality Research Using a Smartphone Dichotic Listening Application. Front Psychol. 2013;4:42 10.3389/fpsyg.2013.00042 23404376 PMC3566356

[20] Behrmann M, Plaut DC. Distributed circuits, not circumscribed centers, mediate visual recognition. Trends in Cognitive Sciences. 2013;17(5):210–9. 10.1016/j.tics.2013.03.007 23608364

[21] Boles DB, Barth JM. "Does degree of asymmetry relate to performance?" A critical review. Brain Cogn. 2011;76(1):1–4. 10.1016/j.bandc.2011.01.013 21371803

[22] Hirnstein M, Hugdahl K, Hausmann M. How brain asymmetry relates to performance—a large-scale dichotic listening study. Front Psychol. 2014;4:997 10.3389/fpsyg.2013.00997 24427151 PMC3877751

[23] Mellet E, Jobard G, Zago L, Crivello F, Petit L, Joliot M, et al Relationships between hand laterality and verbal and spatial skills in 436 healthy adults balanced for handedness. Laterality. 2014;19(4):383–404. 10.1080/1357650X.2013.796965 23745714

[24] Mazoyer B, Mellet E, Perchey G, Zago L, Crivello F, Jobard G, et al BIL&GIN: A neuroimaging, cognitive, behavioral, and genetic database for the study of human brain lateralization. Neuroimage. 2016;124(Pt B):1225–31. 10.1016/j.neuroimage.2015.02.071 25840118

[25] Thiel A, Habedank B, Herholz K, Kessler J, Winhuisen L, Haupt WF, et al From the left to the right: How the brain compensates progressive loss of language function. Brain Lang. 2006;98(1):5765. 10.1016/j.bandl.2006.01.007 16519926

[26] Bishop DVM. Which neuroimaging measures are useful for individual differences research? [Internet]. BishopBlog. 2017 [cited 28 May 2017]. Available from: http://deevybee.blogspot.be/2017/05/which-neuroimaging-measures-are-useful.html?m=1

[27] Van der Haegen L, Cai Q, Stevens MA, Brysbaert M. Interhemispheric Communication Influences Reading Behavior. J Cognitive Neurosci. 2013;25(9):1442–52. 10.1162/jocn_a_00412 23647517

[28] Oregan JK, Jacobs AM. Optimal viewing position effect in word recognition—A challenge to current theory. J Exp Psychol Human. 1992;18(1):185–97.

[29] Kimura D. Functional asymmetry of the brain in dichotic listening. Cortex. 1967;3:163–178.

[30] Dundas EM, Plaut DC, Behrmann M. The Joint Development of Hemispheric Lateralization for Words and Faces. J Exp Psychol Gen. 2013;142(2):348–58. 10.1037/a0029503 22866684 PMC4241688

[31] Rossion B, Caharel S. ERP evidence for the speed of face categorization in the human brain: Disentangling the contribution of low-level visual cues from face perception. Vision Res. 2011;51(12):1297–311. 10.1016/j.visres.2011.04.003 21549144

[32] Tzourio-Mazoyer N, Petit L, Razafimandimby A, Crivello F, Zago L, Jobard G, et al Left hemisphere lateralization for language in right-handers is controlled in part by familial sinistrality, manual preference strength, and head size. The Journal of neuroscience : the official journal of the Society for Neuroscience. 2010;30(40):13314–8. 10.1523/JNEUROSCI.2593-10.2010 PMC6634737 20926657

[33] Oldfield RC. The assessment and analysis of handedness: The Edinburgh Inventory. Neuropsychologia. 1971;9(1):97–113. 5146491 10.1016/0028-3932(71)90067-4

[34] Peters M, Durding BM. Handedness measured by finger tapping: a continuous variable. Can J Psychol. 1978;32(4):257–61. 752415 10.1037/h0081694

[35] Brus B, Voeten M. Een-minuut-test vorm A en B, schoolvorderingstest voor de technische leesvaardigheid bestemd voor groep 4 tot en met 8 van het basisonderwijs Verantwoording en handleiding. Lisse: Swets & Zeitlinger; 1991.

[36] van den Bos K, Spelberg H, Scheepsma A, de Vries J. De klepel vorm A en B, een test voor leesvaardigheid van pseudowoorden Verantwoording, handleiding, diagnostiek en behandeling. Lisse: Swets & Zeitlinger; 1999.

[37] Kuperman V, Van Dyke JA. Effects of individual differences in verbal skills on eye-movement patterns during sentence reading. J Mem Lang. 2011;65(1):42–73. 10.1016/j.jml.2011.03.002 21709808 PMC3119501

[38] Callens M, Tops W, Brysbaert M. Cognitive Profile of Students Who Enter Higher Education with an Indication of Dyslexia. Plos One. 2012;7(6). 10.1371/journal.pone.0038081 PMC3374824 22719864

[39] Lemhofer K, Broersma M. Introducing LexTALE: a quick and valid Lexical Test for Advanced Learners of English. Behav Res Methods. 2012;44(2):325–43. 10.3758/s13428-011-0146-0 21898159 PMC3356522

[40] Willemin J, Hausmann M, Brysbaert M, Dael N, Chmetz F, Fioravera A, et al Stability of right visual field advantage in an international lateralized lexical decision task irrespective of participants' sex, handedness or bilingualism. Laterality. 2016:1–24. 10.1080/1357650X.2015.1047782 26775679

[41] Porac C, Coren S. Lateral preferences and human behavior New York: Springer-Verlag; 1981.

[42] Baayen R, Piepenbrock R, Van Rijn H. The CELEX lexical database. [CD-ROM]. Philadelphia: University of Pennsylvania: Linguistic Data Consortium; 1993.

[43] Keuleers E, Diependaele K, Brysbaert M. Practice effects in large-scale visual word recognition studies: a lexical decision study on 14,000 dutch mono- and disyllabic words and nonwords. Front Psychol. 2010;1:174 10.3389/fpsyg.2010.00174 21833236 PMC3153785

[44] Morris SB, DeShon RP. Combining effect size estimates in meta-analysis with repeated measures and independent-groups designs. Psychological methods. 2002;7(1):105–25. 11928886 10.1037/1082-989x.7.1.105

[45] Cai Q, Van der Haegen L, Brysbaert M. Complementary hemispheric specialization for language production and visuospatial attention. P Natl Acad Sci USA. 2013;110(4):E322–E30. 10.1073/pnas.1212956110 PMC3557046 23297206

[46] Seghier ML. Laterality index in functional MRI: methodological issues. Magnetic Resonance Imaging. 2008;26(5):594–601. 10.1016/j.mri.2007.10.010 18158224 PMC2726301

[47] van den Noort M, Specht K, Rimol LM, Ersland L, Hugdahl K. A new verbal reports fMRI dichotic listening paradigm for studies of hemispheric asymmetry. Neuroimage. 2008;40(2):902–11. 10.1016/j.neuroimage.2007.11.051 18234509

[48] Specht K. Mapping a lateralization gradient within the ventral stream for auditory speech perception. Frontiers in human neuroscience. 2013;7:629 10.3389/fnhum.2013.00629 24106470 PMC3788379

[49] Yovel G, Tambini A, Brandman T. The asymmetry of the fusiform face area is a stable individual characteristic that underlies the left-visual-field superiority for faces. Neuropsychologia. 2008;46(13):3061–8. 10.1016/j.neuropsychologia.2008.06.017 18639566

[50] Hirnstein M, Hausmann M, Gunturkun O. The evolutionary origins of functional cerebral asymmetries in humans: does lateralization enhance parallel processing? Behav Brain Res. 2008;187(2):297–303. 10.1016/j.bbr.2007.09.023 18029034

[51] Brysbaert M, Dydewalle G. Individual Analysis of Laterality Data. Neuropsychologia. 1990;28(9):901–16. 2259423 10.1016/0028-3932(90)90107-y

[52] Hedge C, Powell G, Sumner P. The reliability paradox: Why robust cognitive tasks do not produce reliable individual differences. Behav Res Methods. 2017. 10.3758/s13428-017-0935-1 PMC5990556 28726177

[53] Spearman C. Correlation calculated from faulty data. British Journal of Psychology. 1910;3:271–295.

[54] Bukowski H, Dricot L, Hanseeuw B, Rossion B. Cerebral lateralization of face-sensitive areas in left-handers: Only the FFA does not get it right. Cortex. 2013;49(9):2583–9. 10.1016/j.cortex.2013.05.002 23906596

