# Supplementary figures and images for: The relationship between behavioral language laterality, face laterality and language performance in left-handers
Source: PLoS One. 2018 Dec 21;13(12):e0208696. doi: 10.1371/journal.pone.0208696 (PMC6303078; doi:10.1371/journal.pone.0208696)

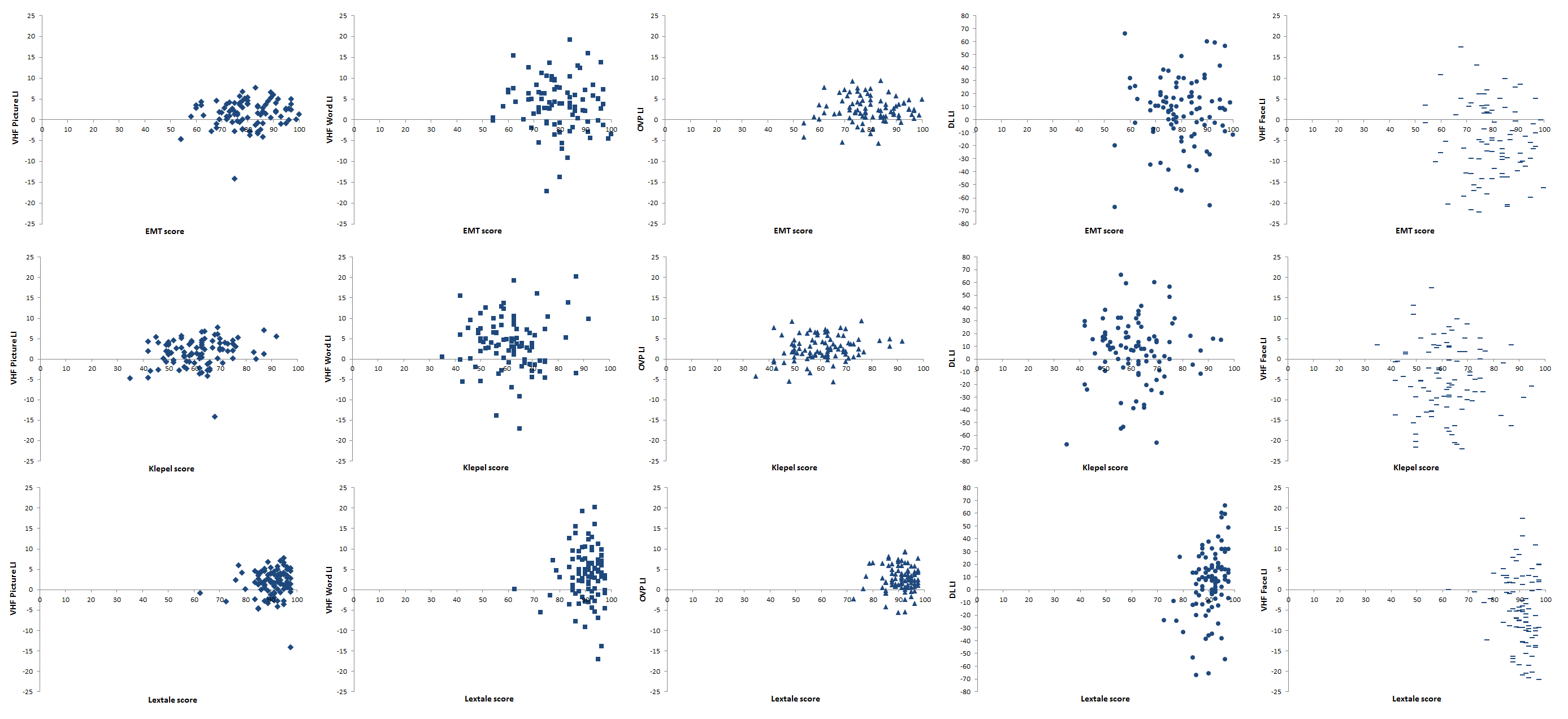

Supplement: S1 Fig — VHF = visual half field; LI = lateralization index; OVP = optimal viewing position; DL = dichotic listening; EMT = One-minute-test. (TIF) [file pone.0208696.s002.tif]
